# Belzutifan as a possible novel treatment for sporadic central nervous system hemangioblastoma: a report of two cases and focused review of the literature

**DOI:** 10.3389/fonc.2026.1935078

**Published:** 2026-10-26

**Authors:** Britta L. Bureau, Ataollah Shahbandi, Saman Shabani, Jennifer M. Connelly

**Affiliations:** 1 Department of Neurology, Medical College of Wisconsin, Milwaukee, WI, United States; 2 Department of Neurosurgery, Medical College of Wisconsin, Milwaukee, WI, United States

**Keywords:** belzutifan, case reports, central nervous system neoplasm, hemangioblastoma, HIF-2α inhibitor

## Abstract

**Objective:**

To provide a review of hemangioblastoma treatment and to report a new treatment option for sporadic, non-von Hippel-Lindau (VHL) associated central nervous system hemangioblastomas (CNS-HBs).

**Background:**

Belzutifan is FDA-approved for the treatment of VHL associated cancers including renal cell carcinoma, pancreatic neuroendocrine tumors and CNS-HBs as a hypoxia-inducible factor-2α inhibitor. The efficacy of belzutifan is unclear for sporadic, non-vHL associated CNS-HBs, a disease entity with limited systemic options.

**Discussion:**

A 23-year-old female presented with a posterior fossa hemangioblastoma which was then resected. At initial recurrence, she completed fractionated radiation therapy with a positive radiographic response. She had multiple symptomatic radiographic recurrences that underwent additional resection. After her fourth recurrence, surgery as well as radiation were not felt to be a reasonable option. Belzutifan 120 mg daily was started. Two months later, she had radiographic improvement with reduction in size of the posterior fossa hemangioblastoma, which has been maintained after 24 months of treatment. A second case is a 33-year-old male with a C7-T1 non-vHL hemangioblastoma that was resected and radiated at recurrence. Nine years later, the patient had radiographic progression that was not amenable to surgery or radiation. Belzutifan was started, which resulted in radiographic and subjective symptomatic improvement.

**Conclusion:**

We present two cases of sporadic recurrent hemangioblastoma with radiographic improvement to belzutifan, along with a review of hemangioblastoma treatment. To the best of our knowledge, these are the first two cases of radiographic improvement in sporadic, non-VHL associated CNS-HB from the use of belzutifan.

## Introduction

Hemangioblastomas are rare and highly vascular tumors that occur within the central nervous system (CNS). These tumors can manifest either sporadically or in association with von Hippel-Lindau (VHL) disease (1, 2). Hemangioblastomas linked with VHL have well-defined genetic mechanisms and established targeted treatment options, whereas sporadic cases pose a significant clinical challenge, as there are currently no comprehensive systemic therapeutic strategies available, aside from surgical resection and radiation therapy (3–6). Belzutifan, an inhibitor of hypoxia-inducible factor-2α (HIF-2α), has been granted FDA approval for the treatment of VHL-associated renal cell carcinoma, pancreatic neuroendocrine tumors, and CNS hemangioblastomas (7, 8). However, its efficacy in the treatment of sporadic, non-VHL hemangioblastomas has not been clearly defined (9). The pathophysiology of sporadic CNS hemangioblastomas is like that of VHL-related hemangioblastomas, so it is theorized that the approach used in VHL hemangioblastomas would be efficacious in the sporadic setting as well.

This report presents two cases of sporadic CNS hemangioblastomas that exhibited radiographic improvement following treatment with belzutifan, after conventional therapeutic options had been exhausted. Both patients provided written consent. Previous studies have indicated that belzutifan can effectively reduce tumor size in VHL-associated CNS hemangioblastomas, yet its role in sporadic cases remains unclear (10, 11). Given that approximately 75% of hemangioblastomas occur sporadically, identifying targeted therapies for this patient population is essential and could have a significant impact on clinical management (2). Our findings provide intriguing preliminary evidence that belzutifan may represent a valuable alternative for patients with progressive or unresectable sporadic CNS hemangioblastomas, thereby warranting further investigation.

## Clinical presentation

### Case report 1: posterior fossa hemangioblastoma

A 23-year-old female initially presented with new onset headache, neck stiffness and imbalance. MRI demonstrated a 4 cm cyst with enhancing nodule in the posterior fossa and resultant hydrocephalus. She underwent resection and pathology confirmed hemangioblastoma WHO Grade 1. She had no family history of Von Hippel-Lindau (VHL) Syndrome. Genetic germline testing (Invitae) for VHL was negative. There were no pathogenic mutations, variants of unknown significance or gross deletions or duplications identified in any of the genes analyzed. Molecular testing was not completed as there was not enough tissue. Systemic imaging was done at the time of diagnosis and was negative. There was no clinical stigmata or family history to support the diagnosis of VHL.

Nine years later, she re-presented with headache, nausea and vomiting. MRI demonstrated a 3 cm local recurrence with hydrocephalus. A ventriculoperitoneal shunt (VPS) was placed. Neuroaxis imaging and CSF studies were negative for drop metastases or CSF involvement. Due to proximity to the brainstem and tumor size, she completed fractionated radiation (54 Gy in 30 fractions) with a positive radiographic response. Six years later, she had an asymptomatic radiographic recurrence for which she underwent a second resection confirming recurrent disease. Post-op imaging showed residual disease along the posterior margin of the brainstem. Four months later, she developed a second lesion involving the right basal frontal region. This was completely resected and confirmed hemangioblastoma. Post-op imaging showed no evidence of residual disease involving the frontal region. Genetic testing for VHL was negative. MRI spine was updated and showed faint superficial leptomeningeal enhancement and intradural, extramedullary enhancement most pronounced in the thoracic cord concerning for drop metastases. CSF analysis including cytology was negative. The patient had multiple surgeries over the past year for recurrence of disease. Residual disease was not amenable to surgery. Given her prior radiation, focused radiation was not ideal due to significant increased toxicity to the brainstem. Given the limited treatment options available for sporadic CNS hemangioblastomas and the progressive nature of the disease despite multiple interventions, systemic treatment options were recommended. While she did not have a clinical diagnosis of VHL, studies have shown that sporadic hemangioblastomas can occasionally carry the HIF2A mutation (9). Therefore, the patient proceeded with a trial of belzutifan 120 mg daily which was well tolerated. Prior to belzutifan treatment the patient had a KPS score of 80 and ECOG of 1 (pre-treatment score). Adverse events were assessed using formal grading via The Common Terminology Criteria for Adverse Events (CTCAE). Regular monitoring included a CBC and pulse oximetry. Anemia, hypoxia and fatigue were a Grade 1 according to CTCAE. Pulse oximetry remained in normal range. Radiographic imaging performed two months after initiation of belzutifan demonstrated improvement (**Table 1**). The fourth ventricle lesion before belzutifan measured 1.6 x 1.6 x 0.8 cm and two months after starting belzutifan measured 1.3 x 1.1 x 0.7 cm (**Figures 1**, **2**). The leptomeningeal enhancement of the thoracic spinal cord also resolved. After treatment the patient has a KPS score of 80 and ECOG of 1 (post-treatment score). There was only one interruption in treatment for a month that was due to a respiratory infection, there was no dose reduction. The patient endorsed being satisfied with having been on the medication for over two years with no significant side effects and has still been able to work full time. The patient remains on treatment with belzutifan.

**Table 1:** Lesion measurement pre and post belzutifan.

Lesion Location	Measurement prior to belzutifan (baseline MRI)	Measurement two months after initiating belzutifan
Fourth Ventricle	1.6 x 1.6 x 0.8 cm	1.3 x 1.1 x 0.7 cm

**Figure 1:**
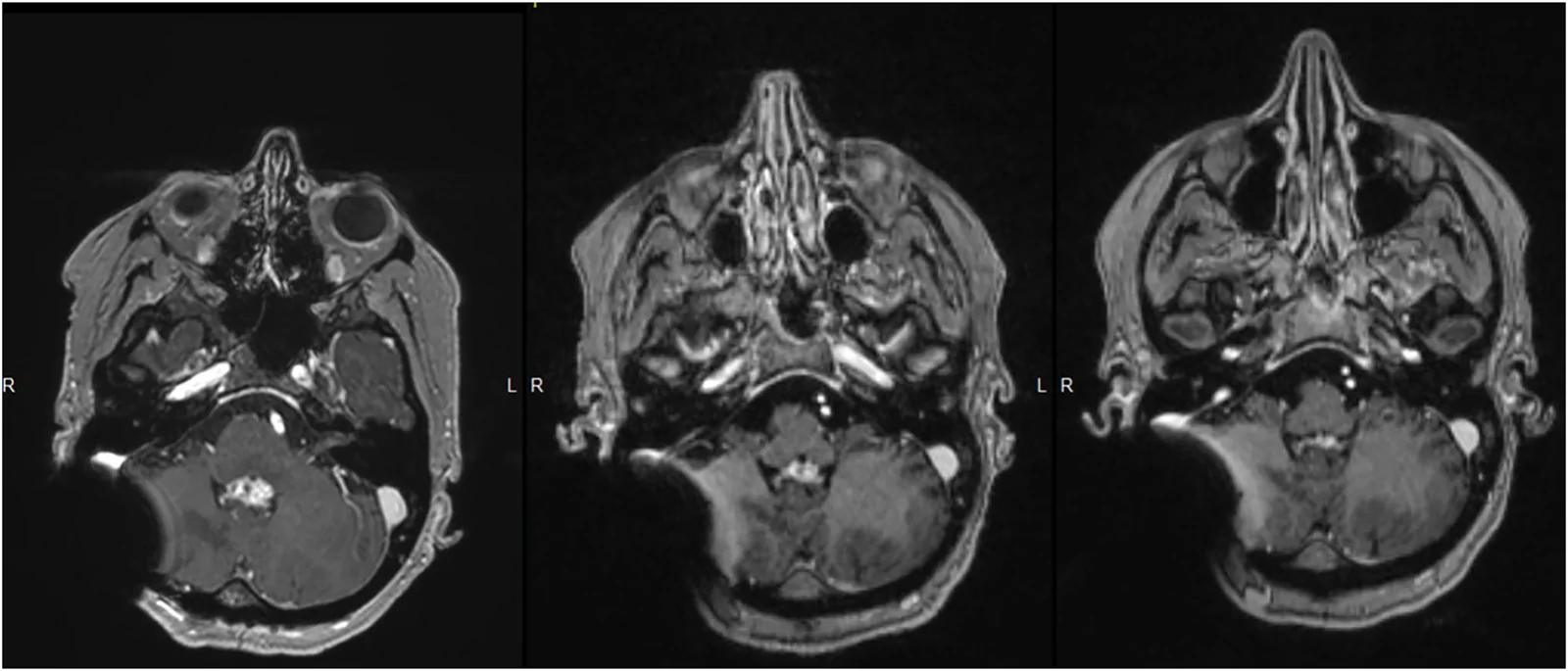
MRI Brain of posterior fossa residual hemangioblastoma pre-belzutifan, two months post-belzutifan, and 24 months post-belzutifan.

**Figure 2:**
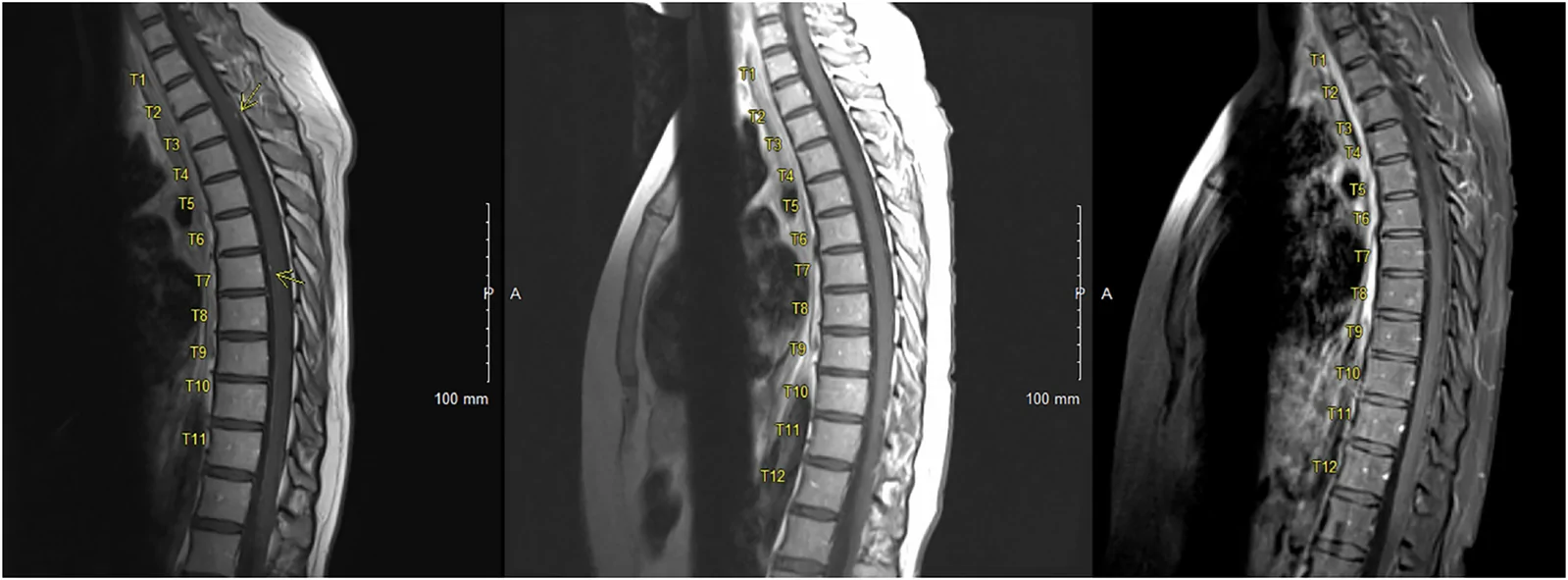
MRI Thoracic spine Left: faint leptomeningeal, intradural and extramedullary enhancement most pronounced from T2–3 through T8-T9; Middle: interval treatment response two months post-belzutifan of intradural, extramedullary leptomeningeal enhancement; Right: unchanged foci of intradural extramedullary enhancement in the thoracic spinal canal at levels of T2 and T4-5, leptomeningeal enhancement no longer identified, 24 months post-belzutifan.

### Case report 2: spinal cord hemangioblastoma

A 33-year-old male presented with left toe numbness that progressed to left leg numbness, inability to move his right foot, numbness and weakness of bilateral hands. On neurological examination, right pupil enlargement was also noted. MRI of the spine revealed a C7-T1 spinal mass that measured 1.1 cm by 2.9 cm. Angiogram revealed vascular abnormality with a small feeding branch from the right vertebral artery and a larger feeding branch from the left costocervical trunk most consistent with a hemangioblastoma. He underwent embolization of the cervical lesion followed by C6 to T2 laminectomy and resection of the mass. Post-op he had an incomplete C6 and spastic quadriplegia. Pathology was consistent with a spinal cord hemangioblastoma WHO Grade 1. Post-op MRI showed persistent tumor. Germline genetic testing (Invitae) was negative for VHL on their Ambry Genetics CustomNext- Cancer + RNA panel genetic test. There were no pathogenic mutations, variants of unknown significance, gross deletions or duplications were identified in any of the genes analyzed. Molecular testing was not completed as there was not enough tissue. Systemic imaging was done at time of diagnosis and was negative. There was no clinical stigmata or family history to support the diagnosis of VHL. Seventeen years after initial presentation, he had a clinical decline which included bilateral leg numbness, left greater than right, along with left hand weakness and numbness with radiographic recurrence. Subsequently, he completed fractionated C-spine radiation (46 Gy in 23 fractions). For symptom management of spasticity, an intrathecal baclofen pump was placed. He went on to have posterior thoracic T5 laminectomy and placement of syringo-subarachnoid shunt that was complicated by an epidural hematoma that needed emergent evacuation. Nine years after the initial recurrence and treatment with radiation, MRI C spine demonstrated slow progression of tumor over several years. Due to location, redo surgery or re-irradiation was not recommended. Bevacizumab was initiated but he had no significant radiographic response or clinical benefit. Therefore, bevacizumab was discontinued. A trial of belzutifan was initiated, which was also tolerated well. Again, regular monitoring included CBC and pulse oximetry. Eight months after initiation of belzutifan, he reported subjective improvement and additionally there was radiographic improvement (**Figure 3**, **Table 2**). Formal response criteria were not applied. The patient has a KPS of 70 or ECOG of 2 (post-treatment score). Patient endorsed that he initially had some mild fatigue and a mild dip in hemoglobin, these symptoms were a Grade 1 according to CTCAE. Overall, he endorses he is feeling much better since starting the belzutifan. This patient never had a dose reduction or interruption. The patient has been able to start feeling both of his legs and hands again, moving his right foot for the first time in five years, more strength in his hands and more energy. He endorsed Belzutifan made a significant improvement in his quality of life. The patient remains on treatment with belzutifan.

**Figure 3:**
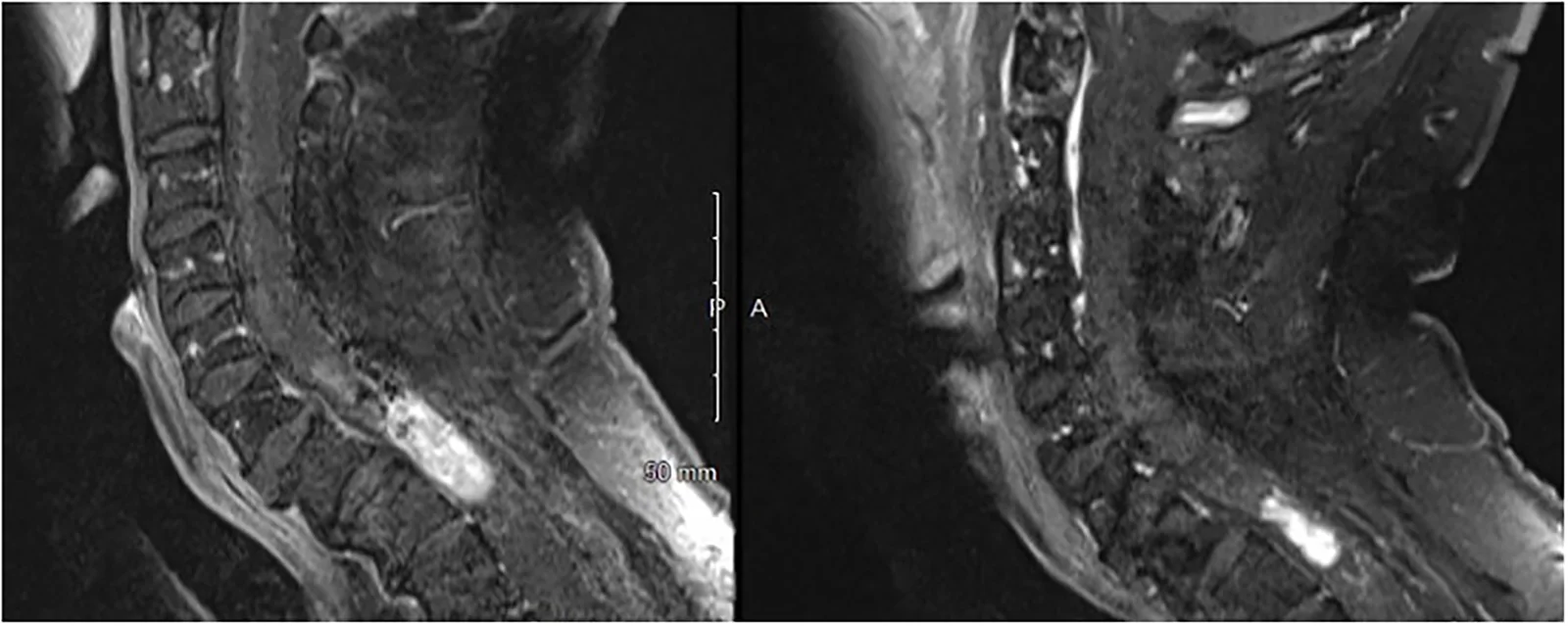
Left: MRI sagittal view post contrast prior to belzutifan initiation 1.1 cm x 2.9 cm; Right: MRI T spine eight months post-belzutifan showing decreased size of enhancing components at C7-T1 levels 0.6 cm x 1.6 cm.

**Table 2:** Lesion measurement pre and post belzutifan.

Lesion Location	Measurement prior to belzutifan (baseline MRI)	Most recent MRI
Spine	1.l x 2.9 cm	0.6 x 1.6 cm

## Discussion

Central nervous system hemangioblastomas (CNS-HBs) are rare, slow-growing, well-demarcated, highly vascularized tumors of the central nervous system (CNS) (9). They most often occur in the posterior fossa (cerebellum and brainstem) or spinal cord (9). CNS-HBs are significant because they comprise 7-10% of posterior fossa tumors in adults and 4% of all spinal cord tumors (12). CNS-HBs can occur sporadically or in association with VHL.

VHL is an autosomal dominant disease that is characterized by multiorgan tumors. These tumors mostly commonly include renal cell carcinoma (RCC), CNS-HBs, endolymphatic sac tumors, and pancreatic neuroendocrine tumors (pNETs). CNS-HBs are present in 60-80% of patients in VHL disease – 45% are in the cerebellum, 36% in the spinal cord and the rest in the brainstem, cauda equina, supratentorium, pituitary stalk, optic nerves and spinal nerve roots (13). VHL is associated with mutation of the VHL gene on chromosome 3 which results in constitutive activation of hypoxia-inducible factors (HIFs) even in normoxia which leads to dysregulated cell growth in multiple tissues (13). There are three distinct alpha subunits on HIFs that have unique functions (13). Experiments have shown that HIF-2alpha leads to aggressive growth and a vascular proliferative phenotype that may drive malignancy in VHL disease (13, 14). This led to the development of a HIF-2alpha inhibitor belzutifan.

Belzutifan is a targeted oral agent that inhibits HIF-2alpha. At this time, belzutifan is FDA-approved for the treatment of VHL associated cancers including renal cell carcinoma, pancreatic neuroendocrine tumors (pNETs) such as pheochromocytomas and paragangliomas (12, 13) and CNS-HBs. Belzutifan’s efficacy for renal cell carcinoma (RCC) with VHL mutations was established in the clinical trial NCT 03401788 and the FDA approved belzutifan for VHL associated RCC, CNS-HBs, and pNETs (7, 8, 13, 15). Of these 61 patients with at least one solid kidney lesion, 39% also had CNS-HBs, and of these patients 63% had a radiographic response even though specific imaging features were not noted in trial results (8, 13). Stevens et al. were the first to report two cases of VHL patients with strong early clinical and radiographic response to belzutifan which included reduction in tumor diameter and perilesional edema of intracranial and spinal hemangioblastomas (13). Zhang et al. demonstrated that VHL-associated hemangioblastomas had a 30% response rate to belzutifan and a 30-50% reduction in their size over the duration of treatment (10). Zamarud et al. did a retrospective study that initiated belzutifan for RCC but noticed that CNS-HBs decreased 30% in size at approximately the four-month mark (11). As evidenced by these studies, it has been clinically proven that belzutifan has been effective at reducing the size of VHL associated CNS-HBs. The most common adverse effects of belzutifan were anemia, headache, renal function impairment, dizziness, and fatigue (13).

Historically treatment of CNS-HBs has depended on whether the CNS-HB is associated with VHL or not. If the tumor is sporadic, then surgical resection and radiation are the treatment options depending on the location of the tumor but there is no FDA approved systemic treatment. There are molecular similarities between sporadic CNS-HBs and VHL associated CNS-HBs, pancreatic neuroendocrine tumors (pNETs) such as pheochromocytomas/paragangliomas (PPGLs), and renal cell carcinoma (RCCs) (9, 13). Taieb et al. found that the Q557* variant of HIF-2A loses its hypoxia sensing function but maintains its ability for transcription which can lead to a pseudohypoxic state even under conditions of normoxia (9). The Q557* mutation of HIF-2A is therefore associated with a decrease in the protein’s oxygen sensing ability (9). Therefore, these tumors are all considered to have a pseudohypoxic state as a result of overexpression of the HIF target genes (9). Taieb et al. present a novel finding that sporadic CNS-HB may be associated with somatic HIF2A mutations. The study screened 28 sporadic HB’s for somatic HIF2A mutations by direct sequencing (9). The patients had no family history of VHL and no personal history of VHL clinical manifestations. Of the 28 patients, two had sporadic CNS-HBs with HIF2A mutations, a 7.1% incidence rate (9). One patient had HIF2A missense mutations, and the other one had a coexistence of somatic mutation in HIF2A and VHL (9). This suggests that HIF2A mutations are not just involved in VHL associated CNS-HBs but also involved in the tumorigenesis of some sporadic CNS-HBs and should be considered a susceptibility gene candidate in sporadic CNS-HBs (9). Therefore, Taieb et al. showed for the first time the presence of HIF2A in sporadic HB. It is thought that the coexistence of HIF2A and VHL genetic abnormalities coalesce to increase tumorigenesis through the over-activation of the HIF signaling pathways and cause a pseudohypoxic phenotype (9). The study concludes that HIF2A should be a considered a gene to target for CNS-HB (9).

Strengths of our study include, to the best of our knowledge, this is the first two cases of radiographic improvement in sporadic, non-VHL associated CNS-HB from the use of belzutifan. There is a concern that VHL Is underdiagnosed and some sporadic CNS hemangioblastomas may be occurring in the setting of undiagnosed VHL or mosaicism so this would standardize treatment of hemangioblastoma. Of note, this manuscript adheres to CARE guidelines. Limitations to our study are that it is a report of two cases. Additional long-term studies with larger cohorts with tissue correlation of HIF2A and prospective clinical trials will need to be done to further assess the effectiveness in belzutifan in managing sporadic CNS-HBs. Additionally, next-generation sequencing of the tumors was not performed so it cannot be definitively determined whether the patient’s response stemmed from an acquired HIF2A mutation in sporadic mutation or less likely from somatic VHL inactivation of the tumor itself. Information on assay sensitivity for low-level VHL mosaicism was not available so this could be a possible limitation. Conducting further studies on biomarkers could be a viable direction for further research to determine which patients would respond to belzutifan. Given there were only two patients reported, safety of belzutifan cannot be established from this study.

## Conclusion

We present two cases as the first documented cases of sporadic, non-VHL associated CNS-HB that have shown clinical and radiographic improvement from the use of belzutifan. Historically, belzutifan has been used to treat VHL associated CNS-HBs, but systemic treatments for sporadic CNS-HBs is limited. Our cases demonstrate that belzutifan exhibits potential efficacy and safety in treating non-VHL associated CNS-HBs, especially those that are recurrent or unresectable. In our cases, belzutifan was notable for radiographic tumor response and minimal side effects. Approximately 75% of HB are sporadic and not related to VHL (12). Having a systemic medication option could alter clinical management and potentially have clinical significance in this non-VHL patient population, additional studies need to be performed.
